# The Porcelain Crab Transcriptome and PCAD, the Porcelain Crab Microarray and Sequence Database

**DOI:** 10.1371/journal.pone.0009327

**Published:** 2010-02-19

**Authors:** Abderrahmane Tagmount, Mei Wang, Erika Lindquist, Yoshihiro Tanaka, Kristen S. Teranishi, Shinichi Sunagawa, Mike Wong, Jonathon H. Stillman

**Affiliations:** 1 Romberg Tiburon Center and Department of Biology, San Francisco State University, Tiburon, California, United States of America; 2 Department of Energy Joint Genome Institute, Walnut Creek, California, United States of America; 3 School of Natural Sciences, University of California Merced, Merced, California, United States of America; 4 Center for Computing in the Life Sciences, San Francisco State University, San Francisco, California, United States of America; 5 Department of Integrative Biology, University of California, Berkeley, California, United States of America; Institute of Genomics and Integrative Biology, India

## Abstract

**Background:**

With the emergence of a completed genome sequence of the freshwater crustacean *Daphnia pulex*, construction of genomic-scale sequence databases for additional crustacean sequences are important for comparative genomics and annotation. Porcelain crabs, genus *Petrolisthes*, have been powerful crustacean models for environmental and evolutionary physiology with respect to thermal adaptation and understanding responses of marine organisms to climate change. Here, we present a large-scale EST sequencing and cDNA microarray database project for the porcelain crab *Petrolisthes cinctipes*.

**Methodology/Principal Findings:**

A set of ∼30K unique sequences (UniSeqs) representing ∼19K clusters were generated from ∼98K high quality ESTs from a set of tissue specific non-normalized and mixed-tissue normalized cDNA libraries from the porcelain crab *Petrolisthes cinctipes*. Homology for each UniSeq was assessed using BLAST, InterProScan, GO and KEGG database searches. Approximately 66% of the UniSeqs had homology in at least one of the databases. All EST and UniSeq sequences along with annotation results and coordinated cDNA microarray datasets have been made publicly accessible at the Porcelain Crab Array Database (PCAD), a feature-enriched version of the Stanford and Longhorn Array Databases.

**Conclusions/Significance:**

The EST project presented here represents the third largest sequencing effort for any crustacean, and the largest effort for any crab species. Our assembly and clustering results suggest that our porcelain crab EST data set is equally diverse to the much larger EST set generated in the *Daphnia pulex* genome sequencing project, and thus will be an important resource to the Daphnia research community. Our homology results support the pancrustacea hypothesis and suggest that Malacostraca may be ancestral to Branchiopoda and Hexapoda. Our results also suggest that our cDNA microarrays cover as much of the transcriptome as can reasonably be captured in EST library sequencing approaches, and thus represent a rich resource for studies of environmental genomics.

## Introduction

A recent addition to the set of model organisms for biomedical research was the Branchiopod crustacean *Daphnia pulex*, whose genome sequencing project has made this organism ideal for studies of both ecotoxicogenomics as well as systems biology [1]–[3]. Comparative genomics approaches to addressing basic questions in biology require appropriate phylogenetic position of comparison taxa. For example, comparative genomic studies in vertebrate animals are possible between multiple mammal, bird and fish species that have had their genomes sequenced. The closest phylogenetic comparison taxa available for *Daphnia* are insects that have had their genomes sequenced [4]. Phylogenetic reconstructions of arthropods based on nuclear markers, mitochondrial markers, and whole mitochondrial genome sequences suggest that *Daphnia* are most closely allied with Malacostracan crustaceans (crabs, shrimp, lobsters) and Hexapoda (insects) [5]–[7]. Mitochondrial genome similarity suggests that Branchiopoda and Malacostraca represent monophyletic sister groups with the closest relatedness to Hexapoda [7]. Phylogenetic reconstruction of all ecdysozoa based on nuclear genes, 18S and 28S ribosomal gene sequences, suggests that Branchiopod crustaceans are more closely related to Hexapoda than Malacostracan crustaceans [8]. The relative phylogenetic position of these groups is important for comparative genomics in order to determine whether insect-*Daphnia* comparisons are less favorable than sequence comparisons between *Daphnia* and Malocostracan taxa [4]. Genome-scale comparisons between multiple crustacean and other arthropod taxa may more accurately resolve the phylogenetic reconstructions between crustacean and hexapod arthropoda based on a small number of nuclear loci or in mitochondrial genomes, which are only maternally inherited.

In comparison to other arthropod species, relatively few genome-level sequencing projects are available for crustacean taxa, although recently several large expressed sequence tag (EST) sequencing projects have yielded relatively large amounts of information on the transcriptomes of several additional crustaceans [9]. In this paper, we present the completion of the large-scale EST sequencing project for one of those species, the porcelain crab, *Petrolisthes cinctipes*, which are well suited organisms for environmental genomic analyses of the impacts of climate change.

Porcelain crabs in the genus *Petrolisthes* are common and diverse inhabitants of intertidal zone and nearshore shallow benthic habitats with over 100 species worldwide [10]. The greatest diversity of *Petrolisthes* occurs in the Pacific Ocean, where roughly 90% of the species are found, with species richness divided equally between the Eastern and Western Pacific coastlines. *Petrolisthes* are distributed along two gradients in physical habitat characteristics, latitudinal and vertical [11]–[15]. In the eastern Pacific *Petrolisthes* species are found in four biogeographic provinces northern and southern temperate, tropical, and in the northern Gulf of California, and within each biogeographic province individual species live in specific vertical zones, from subtidal to upper intertidal zone habitats [16]. Most importantly, these distribution gradients alter the thermal environments in which the crabs live, creating a wide range of habitat temperature extremes, as well as habitat temperature fluctuations. Species living in the upper intertidal zone are eurythermal, experiencing and tolerating higher and lower temperatures during low tide than sympatric subtidal congeners, and upper intertidal species in the tropics experience higher extreme temperatures but smaller thermal ranges than intertidal species in temperate regions [11]–[15].

Porcelain crabs are well adapted to the thermal environments they inhabit [11], [14], [15], and thus present an exciting model system for application of comparative functional genomics in studies of gene expression profiles during thermal acclimation (in the laboratory), acclimatization (in the natural environment), or in response to thermal extremes (heat shock or cold shock) that are characteristic of intertidal zone environments [17], [18]. To facilitate use of functional genomics for ecophysiology [18] we have constructed several EST libraries and sequenced a large number of cloned cDNAs from each library (Table 1). Our initial library construction and sequencing was based on a rather small number of clones from non-normalized libraries [19]. Here we present the construction of large normalized EST libraries (Table 1) and assembly and annotation of both normalized and non-normalized libraries.

**Table 1:** Library generation information.

Library Indentifier	Sub-Library identifier	Sub-Library Tissue Type	Pooled tissue types	Sub-Library # clones	Sub-Library experimental treatment conditions^c^
CAYC^a^	1	Heart		768	1
	2			1,152	
	3	Nerve		768	
	4	Whole Crab		1,152	
	5			1,152	
CAYF^a^	1	Heart		384	1
	2			768	
	3			1,920	
	4	Hepatopancreas		768	
	5			1,152	
	6			384	
	7	Gill		768	
	8			1,536	
	9	Claw muscle		384	
	10			768	
CCAG^b^	1	Pooled from multiple tissues and conditions^d^	Heart, Gill, Whole crab “remains” after heart, gill, and hepatopancreas removed.	47,616	2
			Larvae, freshly molted whole crabs		3
			Whole crabs		4
			Heart		5
			Heart, gill, muscle		6
			Heart, gill, nerve, claw muscle		7

a In CAYC + CAYF there were many different cDNA libraries constructed from seven tissue specific pooled RNA isolates resulting from an array of experiments [19]. These libraries were non-normalized.

b In CCAG there was one library made from an RNA sample that was pooled with equal quantity of RNA per treatment for each tissue. This library was normalized to increase EST diversity.

c Treatment Conditions Descriptions:

1. Field collected crabs that were acclimatized across latitudinal (north-south) and seasonal (winter-summer) gradients, heat shock to 30°C, cold shock to 0°C, acclimated to 8°, 12°, 15°, 18°, 22° and 25°C for 2 to 60 days.

2. Heat 30°C, 4 h (2 h, 4 h, and 6 h 15°C recovery), Cold 2°C, 4 h (2 h, 4 h, and 6 h 15°C recovery), H2O2, 0.5 mM (18 h), CdCl2, 50 µM (24 h), Selenate, 50 µM (24 h), Selenite, 50 µM (24 h), Hypersalinity, 54‰ (18 h), Hyposalinity, 13‰ (18 h), Desiccation (24 h), Hypoxia, 2 h (20 min normoxia recovery), Starvation, 15d (2 h postprandial), Insecticide, 1spray Pyrethrin/200 ml, 5 min (4 h recovery).

3. Acclimated for 1–7 days in San Francisco Bay water (salinity 25–32‰)

4. Acclimated for 1 month to 8°, 15°, 18°, & 25°C

5. Field acclimatized, north-south, winter-summer

6. Acclimated for 1 month to 7°, 19°C

7. Acclimated to 1 month in a thermally fluctuating condition (8:18°C, 12 h:12 h)

d Equal amounts of RNA were mixed from each Pooled tissue/treatment to make the pooled RNA sample used to make the CCAG normalized library.

Our goal is to use these libraries to construct cDNA microarrays for use in profiling transcriptome changes that accompany habitat temperature shifts in the natural environment [18], as well as to develop mechanistic understanding of cellular events that occur during shifts in thermal physiology under laboratory conditions. Because both EST sequencing projects and microarray projects generate large amounts of data, we have adopted the Stanford Microarray Database (SMD) [20]–[24] open source platform developed at the University of Texas, the Longhorn Array Database (LAD) [25] to integrate microarray data with EST sequences, clustered consensus sequences, and homology annotation data. In our database, which we have named the Porcelain Crab Array Database (PCAD, http://array.sfsu.edu), we have added some custom features to the basic architecture of SMD/LAD in order to better integrate sequence data, raw microarray data evaluation, and results from statistical analyses of microarray data.

In total, we generated, assembled and annotated ∼98K high quality ESTs into ∼30K unique consensus sequences representing ∼19K clusters. The results of this project represent the third largest EST sequencing effort for any crustacean, behind the cladoceran *Daphnia pulex* and the White shrimp *Litopenaus vannmei* [9], and has generated the most cDNA sequences for any crab species. The transcript diversity we found in the porcelain crab transcriptome mirrors the results of a much larger EST sequencing effort in *Daphnia pulex* [26], the only crustacean genome that has been sequenced to date.

## Materials and Methods

### Library Construction

Construction of non-normalized EST libraries (named CAYC and CAYF) from a range of tissues in the porcelain crab *Petrolisthes cinctipes* (Table 1) have been described previously [19]. Specimens used for construction of the normalized library (named CCAG) (Table 1) were acclimated or exposed to physiological challenges that included variation in temperature, salinity, hypoxia, starvation, exercise, moult cycle, metals, pesticides and desiccation (Table 1). Total RNA was extracted separately for each tissue type used, including heart, gill, nerve, muscle, and whole crabs, and a pooled total RNA was constructed by mixing equal amounts of total RNA from each of the tissue specific pools. Poly(A)+ RNA was isolated from the pooled total RNA using the Stratagene “Absolutely mRNA” Purification kit. Poly(A)+ RNA purity and quantity was assessed with an Agilent Bioanalyzer. First-strand cDNA was prepared using the Creator SMART cDNA Synthesis Kit (Clontech). 1 ug poly(A)+ RNA, SMART IV Oligo (5′-AAGCAGTGGTATCAACGCAGAGTGGCCATTACGGCCrGrGrG-3′) and a specially designed Evrogen CDS-3M adapter (5′AAGCAGTGGTATCAACGCAGAGTGGCCGAGGCGGCCdT20VN 3′) in the TRIMMER-DIRECT cDNA Normalization Kit (Evrogen) were used for first-strand cDNA synthesis.

To prepare cDNA for normalization, first-strand cDNA was amplified by long-distance PCR (LD-PCR) with using the Creator SMART cDNA Synthesis Kit and Advantage 2 PCR Kit (Clontech). Fifteen PCR cycles of 94°C for 7 s, 66°C for 30 s, and 72°C for 6 min were performed. Normalization was performed using the TRIMMER-DIRECT cDNA Normalization Kit (Evrogen). 1 ug of amplified cDNA was purified with Qiagen PCR Purification Kit, precipitated with ethanol and dissolved in nuclease free water. cDNA was mixed with 4X hybridization buffer, overlaid with mineral oil, denatured at 98°C for 3 min and allowed to renature at 68°C for 5 hours. Duplex-specific nuclease (DSN) treatment was performed as described in the Evrogen kit manual. To amplify the ssDNA fraction remaining after DSN treatment, post amplifications were performed by using M1 (5′AAGCAGTGGTATCAACGCAGAGT3′) and M2 (5′AAGCAGTGGTATCAACGCAG3′) primers for eighteen cycles of 94°C for 7 s, 66°C for 30 s, and 72°C for 6 min.

Asymmetric SfiI restriction enzyme sites (SfiI A and SfiIB) were incorporated at the 5′ and 3′ ends of normalized cDNA. After digestion with SfiI and size fractionation (>0.5 kb), normalized cDNA was directionally ligated into SfiI-digested pDNR-LIB vector. The ligation mixture was transformed into ElectroMax T1 DH10B cells by electroporation, and plated on selective media for colony picking into 384-well plates. A total of n =  47,616 colonies (124×384-well plates) were picked and sequenced from both ends on ABI 3730 instruments (Applied Biosystems) using primers pDNR-LIB-seq-Fw2: 5′AGTCAGTGAGCGAGGAAG3′, and pDNR-LIB-seq-Rev: 5′AGGAAACAGCTATGACCAT3′. These primers were used to PCR amplify cloned cDNAs for printing microarrays, and after agarose gel electrophoresis we assessed insert size of about 8000 clones.

Raw EST sequences were trimmed of vector and adaptor sequences based on common sequence patterns at the ends of the sequences. Insertless clones were identified using the following criteria: 200 bases or more are vector masked from the 5′ end of the EST or less than 200 bases of vector masked sequence followed by less than 100 bases of non-vector masked sequence.

ESTs were trimmed for quality using a sliding window trimmer (window  = 11 bases). Once the average quality score in the window was below a threshold score of 15, the EST was split and the longest remaining sequence retained as the trimmed EST. EST sequences with less than 100 bases of high quality sequence were removed. ESTs were evaluated for the presence of a polyA or T tail near the ends and if found then the EST was flagged and the polyA/T tail was trimmed off. ESTs were filtered for length a second time and any ESTs less than 100 bases or with low complexity sequence greater than the threshold of 50% were removed. When the same clone was resequenced, the longest high quality ESTs were retained. If one EST from a sister or end pair of ESTs was insertless or a contaminant then by default the second sister was assigned to the same category. Alternatively each sister read was treated separately for the low complexity and low quality categories. In order to identify and screen out contaminants, BLAST was used to compare the EST sequences against the Genbank nucleotide database. ESTs found to match non-desirable sequences (e.g., non-cellular) were removed.

### Assembly and Clustering

Trimmed high quality ESTs were used in assembly and clustering. The ESTs generated in this project were assembled and clustered a total of four times: In assembly # 1 the non-normalized tissue-specific libraries CAYC and CAYF were assembled and clustered together, as has been previously reported [19]. In assembly #2 the normalized mixed tissue library CCAG was clustered. For assemblies #3 and #4 all available sequence data (libraries CAYC, CAYF and CCAG) were assembled and clustered together. Assemblies #2 and #3 were performed at the Joint Genome Institute as follows. ESTs were clustered using Malign, a kmer based alignment tool that clusters ESTs based on sequence overlap (kmer  = 16, seed length requirement  = 32 alignment ID ≥98%). Malign software [27], is a modified version of the Smith–Waterman algorithm [28], which was developed at the JGI for use in whole-genome shotgun assembly. Clusters of ESTs were further merged based on sister reads using double linkage. Double linkage requires that 2 or more matching sister ESTs are in each cluster to be merged. EST clusters were assembled using CAP3 [29] to form consensus sequences. CAP3 parameters used were the default parameters for the 12/21/07 software version (-a 20, -b 20, -c 12, -d 200, -e 30, -f 20, -g 6, -h 20, -I 40, -j 80, -k 1, -m 2, -n -5, -o 40, -p 90, -r 1, -s 900, -t 300, -u 3, -v 2, y 100, -z 3). Clusters may have more than one consensus sequence for various reasons (e.g., clone has long insert, clones are splice variants, consensus sequences are erroneously not assembled). Cluster singlets were clusters of one EST whereas CAP3 singlets are ESTs that joined a cluster with other ESTs but upon assembly by CAP3 a single EST was used to make a separate consensus sequence from the others. ESTs from each separate cDNA library were clustered and assembled separately and subsequently the entire set of ESTs for all cDNA libraries were clustered and assembled together. For cluster consensus sequence annotation, the consensus sequences were compared to Swissprot using BLASTx, as described below.

Assembly #4 was performed using the EST2uni software pipeline [30] hosted at UC Merced. The resulting assembly and annotation data (see below) are available online (http://sequoia.ucmerced.edu/PetrolESTes/index.php). RepeatMasker (http://www.repeatmasker.org) and seqclean (http://compbio.dfci.harvard.edu/tgi/software/) were run by default to mask low-complexity regions (settings: -s -norna -species eudicotyledons -gccalc –xsmall, seqclean: -n 10 -N -A -L 2) and repetitive elements, and to remove unexpected vector sequences (settings: -n 10 -N -A -L 2). EST clustering was performed by CAP3 [29] with option –p set to 90 (default = 75) for increased assembly stringency. All UniSeqs were auto-BLASTn aligned resulting in clusters of similar UniSeqs.

### Homology Searches and Other Analyses

All of the unique consensus sequences or contigs (Assemblies #1–4) were functionally annotated by BLASTx searches [31]. Using BLASTx with an E-value cutoff of 10^−5^ we compared the porcelain crab transcriptome using BLASTx vs. four databases, GenBank (assembly #1–3), SwissProt (assembly #1–4), UniProt [32] (assembly #1–4), and the *Daphnia pulex* transcripome (FrozenGeneCatalog_2007_07_03.aa.fasta, file available at the JGI genome portal page for *D. pulex*) (assembly #3). We also used tBLASTx vs. the *Daphnia pulex* transcripome (assembly #3) with an E-value cutoff of 10^−5^ and used InterProScan (assembly #3) using 13 different homology search programs (blastprodom, fprintscan, pfam, pir, panther, tigr, smart, superfamily, gene3d, scanregexp, profilescan, seg, coils, tm, signalp, GO). GO and EC classifications were extracted from InterProScan matches.

In assembly #4, unique sequences (UniSeqs) were subjected to open reading frame prediction by ESTScan [33], and we identified short-sequence-repeat microsatellites and sequence variations using Sputnik (http://espressosoftware.com/pages/sputnik.jsp) and local algorithms [30] respectively. Further annotations included HMMER (http://hmmer.janelia.org) searches in pfam [34] and GO-term associations [34] based on the best SwissProt BLASTx hit.

For ortholog assignment and pathway mapping, we submitted the clustered consensus sequences (assembies #1–4) to the KAAS-KEGG Automatic Annotation Server [35]. To assign orthologs, we used the single-directional best-hit option (SBH), using the following combined set of 10 organisms: *hsa (Homo sapiens)*, *mmu (Mus musculus)*, *gga (Gallus gallus)*, *xla (Xenopus laevis)*, *xtr (Xenopus tropicalis)*, *dre (Danio rerio)*, *spu (Strongylocentrotus purpuratus)*, *nve (Nematostella vectensis)*, *dme (Drosophila melanogaster)*, *dpo (Drosophila pseudoobscura)*, *sce (Saccharomyces cerevisiae)*, and *cel (Caenorhabditis elegans)* and a BLAST threshold score of 40. The *Daphnia pulex* v1.0 FrozenGeneCatalog_2007_07_03 (ver 1) transcripts data set was downloaded from the JGI genome portal and submitted to the KAAS-KEGG site using the same parameters.

For reciprocal tBLASTx analyses between *P. cinctipes* and other species, unigene datasets for *Drosophila melanogaster* (Build #64), *Apis mellifera* (Build #6), *Daphnia pulex* (Build #1), *Homo sapiens* (Build #219), *Strongylocentrotus purpuratus* (Build #18), *Anopheles gambiae* (Build #41), *Tribolium castaneum* (Build#12), *Nematostella vectensis* (Build#1), and the SwissProt protein database were downloaded from NCBI on Sep. 14, 2009 from the UniGene database at NCBI. BLASTx (against SwissProt) and tBLASTx (against above-listed unigene databases) were performed using the contig sequences from *P. cinctipes* (assembly #4), and parsed with a bit score cutoff  = 40.

A three-way reciprocal tBLASTx was performed for the combinations *Petrolisthes cinctipes* (assembly #4), *Daphnia pulex*, and *Drosophila melanogaster* or *P. cinctipes, D.pulex* and *Apis mellifera*. In an all-against-all BLAST search (tBLASTx with option -F “m S”, bit score-cutoff = 40), clusters of sequences were considered as reciprocal hits if the best tBLASTx hits for a given sequence against the two other databases were also the best reciprocal tBLASTx hit against each other.

We estimated the number of full-length transcripts using the contigs, but not singletons, from assembly #4 that had strong BLASTx hits vs. SwissProt (bitscore >40) to unique proteins. We determined the residue where the alignment start site occurred and determined whether the sequence length of each contig query was adequate to cover the entire length of the full-length transcript. To do this we parsed the query and hit nucleotide lengths from the BLASTx best alignment, determined the orientation of the query sequences, parsed the query start sites (nucleotide) and hit start sites (amino acid), converted the amino acid sequence into nucleotide lengths, and calculated the number of base pairs missing to the hit start site and hit end site. Then we determined whether the query length would theoretically cover the hit full-length coding sequence by calculating whether it was long enough to extend beyond the first or last residue of the hit. The calculation of percentage of hits that were full length was performed separately for ribosomal protein transcripts and non-ribosomal transcripts.

## Results and Discussion

### cDNA Insert Size, EST Sequencing, Assembly, and Clustering Statistics

Approximately 20% of the cloned cDNAs in the normalized library CCAG yielded PCR products larger than 2 kb, 55% were between 1–2 kb, and 25% were smaller than 1 kb, a similar size distribution from non-normalized libraries CAYC and CAYF [19]. Approximately 122K ESTs were generated from 61K cloned *Petrolisthes cinctipes* cDNAs (Table 2) from three main libraries (Table 1), two non-normalized (CAYC, CAYF) and one normalized (CCAG). The total number of high quality ESTs was ∼98K (Table 2), and these sequences have been deposited at GenBank (accession numbers: FE742652–FE840457). The mean trimmed high-quality EST length was from 500–650 bp, depending on library (Table 2). The non-normalized libraries had a similar number of insertless clones to the normalized library, but a much higher percentage of contaminated clones (Table 2). Thus, a higher fraction of clones from the normalized library were passed on to clustering.

**Table 2:** EST sequencing statistics.

Library	Attempted ESTs	Attempted Clones	Insertless Clones (%)	Contaminated Clones (%)	Clones Passing to Clustering (%)	ESTs Passing to Clustering (%)	Mean trimmed EST length (± 1 SD)
CAYC	10,752	5,376	20 (0.4)	421 (7.8)	4,508 (83.8)	7,727 (71.9)	656.8±165.8
CAYF	16,896	8,448	180 (2.1)	1,139 (13.5)	6,702 (79.3)	12,214 (72.3)	512.5±170.2
CCAG	94,847	47,616	419 (0.9)	209 (0.4)	43,548 (91.5)	77,865 (82.1)	614.8±174
CAYC+CAYF+CCAG	122,495	61,440	619 (1.1)	1,769 (7.3)	54,758 (84.9)	97,806 (79.8)	

Clustering of ESTs performed by the JGI resulted in a total of ∼19K clusters represented by assembly of ESTs into ∼31K unique sequences (UniSeqs) (Table 3). About 23% of the clusters contained one EST, 50% contained two ESTs from the same clone, and 31% of the clusters contained ESTs from more than one clone that were assembled into a consensus sequence, or contiguous sequence (contig) (Table 3). The normalized library contained a much greater percentage of clusters with ESTs from more than one clone (32% vs 23–24%, Table 3), which is likely due to the deeper sequencing of the normalized library. Clustered consensus sequences, or contigs, are archived at the Porcelain Crab Array Database (http://array.sfsu.edu).

**Table 3:** Joint Genome Institute clustering statistics.

Library	Number of clusters	Number of contigs	Clusters with one EST (%)	Clusters with one clone (%)	Clusters with > one clone (%)
CAYC	2,583	3,461	689 (26.7)	1,307 (50.6)	587 (22.7)
CAYF	2,629	3,058	553 (21.0)	1,449 (55.1)	627 (23.8)
CCAG	16,309	26,625	3,703 (22.7)	7,360 (45.1)	5,246 (32.3)
CAYC+CAYF+CCAG	19,312	30,764	4,504 (23.3)	9,709 (50.3)	6,001 (31.1)

Clustering of ESTs performed by the EST2uni-pipeline [30] resulted in a slightly smaller number of ESTs passing quality due to the higher stringency of the set parameters (Table 4). Only 0.2% of ESTs that passed quality in the JGI assembly (Table 2) did not pass quality in the EST2uni assembly (Table 4). A total of ∼28K unique sequences were generated, about half of which singletons derived from one clone, and half of which were contigs derived from more than one clone (Table 4). Thus, the number of unique sequences with EST2uni (Table 4) was ∼2.5K less than for the JGI assembly (Table 3). The average number of ESTs per contig was 5.7, with a maximum of 1,267. UniSeq length ranged from 101 to 4,579 bp, with an average length of 816.5 bp (Table 5). The UniSeqs were clustered into a total of ∼23K clusters by EST2uni (Table 4), ∼3.5K more clusters than from the JGI analysis (Table 3), a result likely to be related to higher clustering stringency used in assembly #4 (see Methods).

**Table 4:** EST2uni assembly and clustering statistics.

Library	ESTs passing quality (%)^a^	UniSeq Type	Number (%)	Cluster of UniSeqs	Number (%)
CAYC	7710 (99.8)	Contig	14,694 (52)	= 1 UniSeq	20,554 (89)
CAYF	12197 (99.9)	Singleton	13,693 (48)	>1 UniSeq	2,458 (11)
CCAG	77741 (99.8)	Total	28,333	Total	23,012
CAYC+CAYF+CCAG	97648 (99.8)				

a The starting set of ESTs was the ESTs Passing to Clustering from the JGI (Table 2).

**Table 5:** UniSeq length distribution, based on EST2uni assembly.

Sequence Length		Number of UniSeqs
From (bp)	To (bp)	
101	548.8	5713
548.8	996.6	15830
996.6	1444.4	4604
1444.4	1892.2	1350
1892.2	2340	554
2340	2787.8	180
2787.8	3235.6	60
3235.6	3683.4	29
3683.4	4131.2	10
4131.2	4579	3

### Potential Molecular Markers

We found a large number of potential molecular markers in assembly #4, including 20,901 ESTs with single nucleotide polymorphisms (SNPs) of which 5,907 were transversions and 14,994 were transitions, and 4,094 ESTs with simple sequence repeats (SSRs) grouped into 39 different sequence repeat motifs, 26 tetranucleotide repeats, 10 trinucleotide repeats, and 3 dinucleotide repeats. The 7 most common repeats making up 74% of the total observed SSRs were AC (24%), AAC (13%), AAT (12%), AG (10%), ACAT (5%), ACT (5%) and ACC (5%). Most SSRs (77%) were observed in 3′ or 5′ untranslated regions (UTRs).

### Annotation

A total of 66% of our cloned cDNAs had strong homology (expect <1e-5) to known sequences in at least one database and search algorithm combination (Table 6). In general we used the expect score cutoff threshold but in the BLASTx vs. SwissProt analysis using assembly #4 we also used a bitscore <40 cutoff and found that the number of strong matches was very similar (Table 6). Of the clones containing ESTs that clustered in contigs with at least one match, 13% matched in all six homology searches, 50% matched in five of six searches, 11% matched four of six searches, 9% matched three of six searches, 6% matched two of six searches, and 11% matched in only one of six searches. The fact that 26% cloned cDNAs were represented by contigs that had strong matches in only one, two or three of the homology searches is why two thirds of the total library is annotated when no individual search returned more than 40% strong homology (Table 6). Overall, of the clones with strong homology, 74% can be considered robust matches, as from four to six homology search method-database combinations returned matches, thus about 49% of our library has robust annotation, about 17% has annotation that likely will require further analysis, and 34% has no annotation.

**Table 6:** Homology search summary.

Homology Search Algorithm	Assembly (query)	Database (date of database searched)	# queries	# Strong Matches (≤1e^∧^-5)	Percentage strong match
BLASTx	Assembly #1,2	GenBank_v159_aa.fasta (July, 2007)	33,114	12,242	36.97%
BLASTx	Assembly #1,2	uniprot_sprot_fasta (September, 2007)	33,114	10,101	30.50%
BLASTx	Assembly #4	uniprot_sprot_fasta (September, 2008)	28,333	7,973^b^	28.14%
InterProScan	Assembly #1,2	blastprodom, fprintscan, pfam, pir, panther, tigr, smart, superfamily, gene3d, scanregexp, profilescan, seg, coils, tm, signalp, GO (October, 2007)	33,114	8,641	26.10%
BLASTx	Assembly #3	daphnia.filtered_models_v1.1.aa.fasta (January, 2008)	30,764	2,997	9.74%
tBLASTx	Assembly #3	FrozenGeneCatalog_2007_07_03.na.fasta (September, 2008)	30,764	8,939	29.06%
Total^a^		BLASTx, tBLASTx, and InterProScan	55,641	36,544	65.68%

a Clones with homology search match in at least one of the algorithms and databases used.

b The number of hits with bitscores >40 was 7,737 (27.31%)

BLASTx searches vs. the GenBank non-redundant protein database yielded the greatest number of strong matches, with about 37% of the porcelain crab transcripts being well represented (Table 6). Of the clones that had only one homology method-database match (see above), 35% matched by BLASTx vs. the nr database. In many cases, these matches were to hypothetical proteins, unknown proteins, or patented sequences with no description. Those types of sequences are not likely to be included in the SwissProt databases, nor indexed by InterProScan algorithm databases, and thus would only be observed in the nr database.

BLASTx vs. SwissProt gave similar results for most of the strong homology matches with both sets of contig sequences. However, in comparison of results from the 2007 and 2008 updates of the UniProt database, we observed a greater percentage of matches in the 2007 search (Table 6), but a much greater number of homology matches during the 2008 search that had no homology in other method-database combinations. Only 1% of the clones that had only one homology result come from the 2007 BLASTx vs. Swissprot analysis, whereas that number is 30% in the 2008 UniProt search. The BLASTx analyses we performed against the GenBank non-redundant and the SwissProt databases resulted in a greater overall percentage of strong matches than the initial homology searches done for *Petrolisthes cinctipes* ESTs [19]. Characterization of the unique consensus sequence set from our initial non-normalized libraries CAYC+CAYF yielded approximately 30% and 23% strong matches (<1e-4) by BLASTx against the GenBank nr and SwissProt databases, respectively [19]. Here, with a more stringent “strong match” qualifier of anything with an expect score of <1e-5, we had about 7% more strong matches for both databases searched (Table 6). This increase may be due to the fact that our normalized and more diverse library CCAG is so much larger than the non-normalized libraries used in our initial homology searches [19].

One possible reason why the BLASTx vs. SwissProt (UniProt) results were different between the 2007 and the 2008 analyses could be that many sequences were added to the UniProt database during the year between our analyses, including those from *Daphnia pulex* (J. Colbourne, pers. comm.). However, if that were the case, then we would expect a greater overall number of matches in the 2008 analysis, which was not what we observed (Table 6). Instead, our results suggest that the EST2uni assembly may have produced some alternative contigs that resulted in alternative results from BLASTx analysis. The fact that the EST2uni UniSeq set was only used in the BLASTx search against the 2008 UniProt database (Table 6) would explain why such a large fraction of the clones with only one homology match resulted from this analysis.

**Figure 1:**
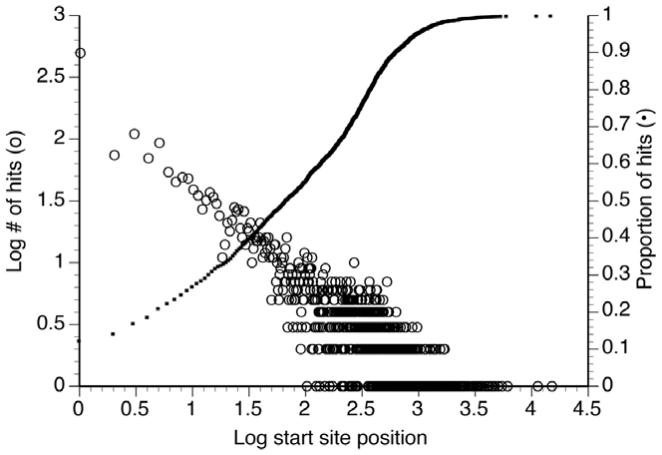
Analysis of BLASTx hit start position for contigs of assembly #4 with unique strong hits. Contigs (n = 3,993) that matched unique genes in the SwissProt database with bitscores >40 are plotted as the number of hits per hit amino acid start site (open symbols) and fractional total of hits (solid symbols).

The number of full-length transcripts in our unigene set was determined using the EST2uni (assembly #4) homology search results. Of the 14,694 contigs (Table 4), 5,869 had bit scores >40, 3,993 of which matched unique genes. Out of those 3,993 contigs, >50% had an alignment start prior to residue 68, and >25% had an alignment start prior to residue 10, with about 12% starting on residue 1 (Figure 1). Full-length transcripts, determined as transcripts that were long enough to theoretically cover the hit sequence completely, were 53.3% of the 3,993 unique contigs, with 4.2% being ribosomal protein genes and 49.5% non-ribosomal protein genes. Because low-quality sequence information was trimmed from both the beginning and end of ESTs, the actual number of full-length cloned cDNAs in our libraries may be greater than represented in our analysis of those 3,993 contigs.

**Figure 2:**
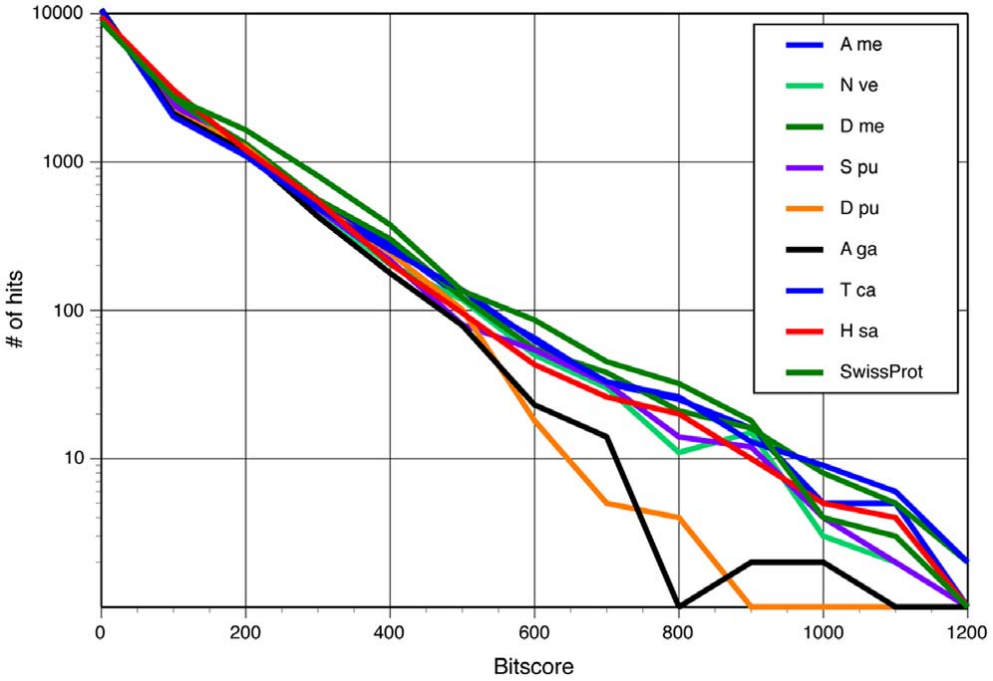
Paired BLASTx homology search results between *Petrolisthes cinctipes* and either the SwissProt uniprot database, or the unigene set of a range of species. (A me  =  *Apis mellifera*, N ve  =  *Nematostella vectensis*, D me  =  *Drosophila melanogaster*, S pu  =  *Strongylocentrus purpuratus*, D pu  =  *Daphnia pulex*, A ga  =  *Anopheles gambiae*, T ca  =  *Tribolium castaneum*, H sa  =  *Homo sapiens*).

BLASTx homology searches against the *Daphnia pulex* protein databases did not produce significantly better homology search results than searches of combined species databases (Table 6) or in unigene protein datasets for other animals with sequenced genomes (Figure 2). Relatively lower amounts of very strong matches (bitscores between 500–1000) were observed in comparisons between *P. cinctipes* and either *D. pulex* and *A. gambiae* (Figure 2) suggesting either that there is lower homology of strongly conserved proteins (where strong blast hits would be), or that the *D. pulex* and *A. gambiae* unigene protein datasets are incomplete relative to other metazoan groups sampled. However, in some cases we obtained significant homology against the *D. pulex* genome database for contigs that had no homology in any of our other method-database combinations. We observed a greater number of strong matches when tBLASTx was used to compare the *P. cinctipes* and *D. pulex* sequence databases. In cases where BLASTx returned a matched *D. pulex* protein ID, this same protein ID also was retrieved by the tBLASTx search, which was a good confirmation that protein and nucleotide level searches were giving comparable results. About 29% of the porcelain crab transcripts translated into a strong match for one of the translated *D. pulex* transcripts. One potential reason for why a lower than expected number of porcelain crab sequences yielded strong matches when compared to the Daphnia databases by BLAST comparisons is that the size of the *Daphnia* gene databases is small compared to the size of the nr or swissprot databases. Thus, a different expect value cutoff or score cutoff may be appropriate for these comparisons. We chose to retain the E-value cutoff of 10^−5^ as this is standard practice in BLAST searches. In our BLASTx analyses vs. the SwissProt database parsing the results as hits with bitscore ≥40 yielded less than 1% fewer strong matches as compared to parsing the result set with the E-value cutoff of 10^−5^ (Table 6). Because sequence databases are continuously being updated and improved we fully expect that BLAST analyses vs. the *D. pulex* unigene sets will produce increased strong matches in future analyses.

InterProScan results were generally in accord when strong matches were obtained with BLASTx searches. However, where BLASTx scored weak or no matches, InterProScan often returned different gene homology results. Because InterProScan search algorithms include homology to aspects of proteins beyond primary sequence, including domain architecture, protein family, and predicted folding patterns, it is possible the some of the InterProScan homology results allow protein classification when BLAST does not. The InterProScan searches did not return a greater number of homologies than did BLASTx or tBLASTx searches. They did, however, return homology matches for some consensus sequences that did not have any strong matches with any of the BLAST homology searches.

**Figure 3:**
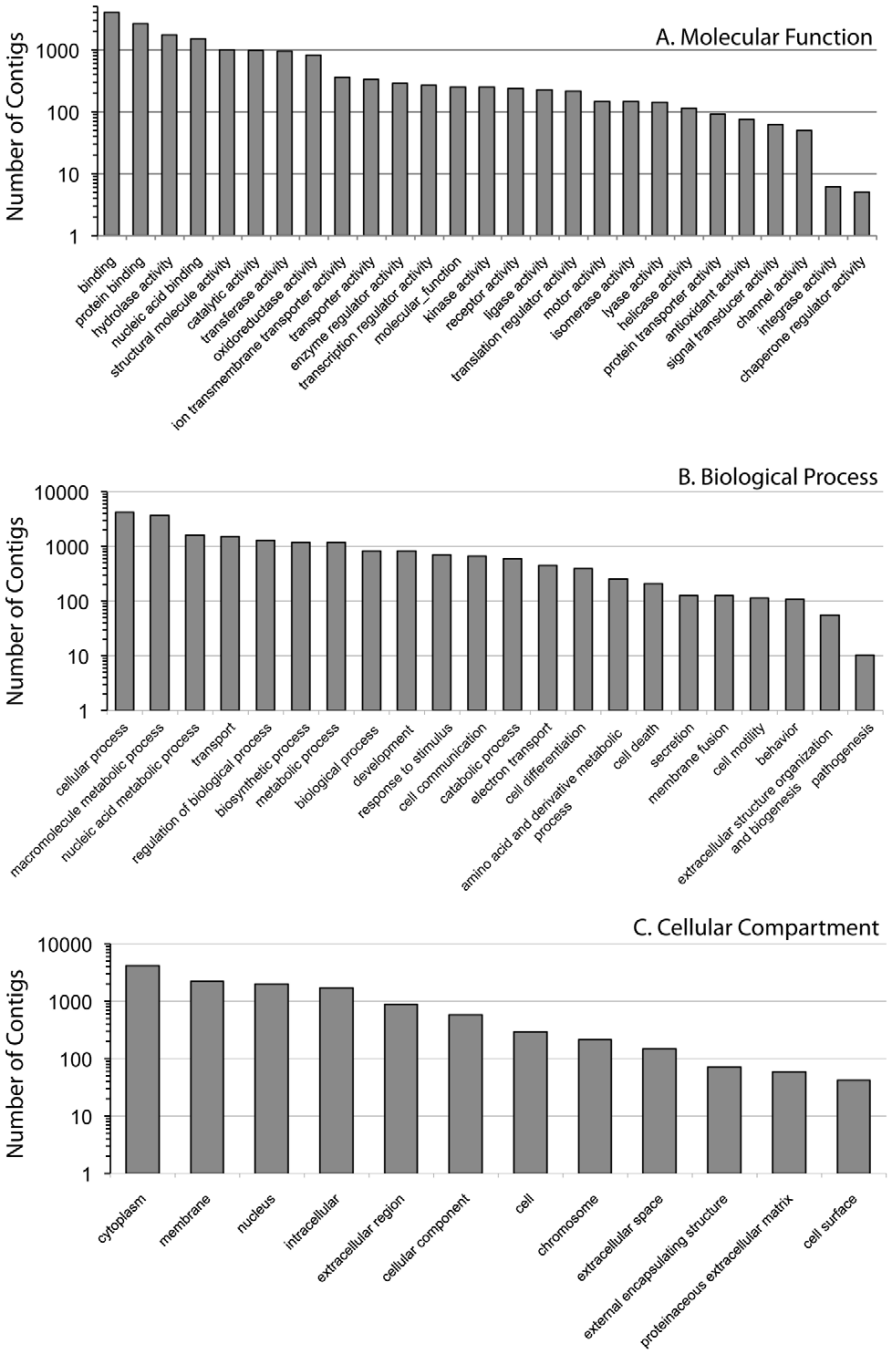
Frequency distributions of condensed Gene Ontology terms. Terms described for A) molecular function, B) biological process, and C) cellular compartment returned in our analyses of the *Petrolisthes cinctipes* transcriptome. A complete list and abundance of GO terms for each ontology, is given in Table S1.

Gene Ontology (GO) analyses were performed based on BLASTx homology search results from the SwissProt Uni-Prot database and from GO terms extracted from InterProScan results from each EST. From InterProScan results, a total of 74,223 matches to 536 unique GO terms for molecular function were returned in our analyses (Table S1). From BLASTx results, a majority of molecular function ontology results (≥1000 contigs) were for binding, especially protein and nucleic acid binding, and for hydrolase, catalytic, transferase, oxidoreductase, structural molecule, and ion transporter activity (Figure 3A). A total of 42,966 matches to 356 unique GO terms for biological processes were returned in our InterProScan analyses (Table S1). Biological processes that were highly represented in our BLASTx GO analyses included cellular and macromolecular metabolic processes (Figure 3B). InterProScan analyses returned 25,173 matches to 131 unique GO terms for cellular compartment (Table S1). From BLASTx analyses the majority of the GO annotated transcripts were compartmentalized in the cytoplasm, membrane, or nucleus (Figure 3C). In general, the results of our GO term analyses suggest that our EST libraries represent a diverse gene set in terms of all three GO categories.

Pathway mapping of clustered consensus sequences through the KEGG KAAS resulted in a total of 215 pathways represented by our consensus sequence set. At the broadest categorical level, our library covers 120 metabolic pathways, 16 genetic information processing pathways, 18 environmental information processing pathways, 32 cellular process pathways, and 29 disease pathways. In all cases, pathways or enzyme classifications for prokaryotic or plant-specific genes were absent in the KAAS results. For specific pathways within each broad category, we had varying amounts of each pathway represented in our gene set. Importantly, our porcelain crab library covers 100% of conserved central energy metabolism pathways of glycolysis, the Krebs cycle, and oxidative phosphorylation (Table 7). Other pathways such as lipid metabolism, nucleotide and protein synthesis pathways, and many signaling pathways (e.g., MAPK, Wnt) were also well represented in our gene set.

**Table 7:** Selected subset of KEGG KAAS Pathway search results for pathways with high coverage.

Pathway ID	Pathway Name	Number of KEGG enzymes in pathway^a^	Number of pathways enzymes in *P. cinctipes*	Percentage of Pathway reactions covered by our library^b^
00010	Glycolysis/Gluconeogenesis (and pyruvate fermentation)	64	23	100%
00020	Citric acid cycle	50	21	100%
00030	Pentose phosphate pathway	42	12	93%
00190	Oxidative phosphorylation	200	88	100%
00071	Fatty acid metabolism	34	18	100%
00230	Purine metabolism	203	46	70%
00240	Pyrimidine metabolism	150	37	80%
00280	Valine, leucine and isoleucine degradation	52	24	76%
03010	Ribosome	147	78	75%
03022	Basal transcription factors	26	16	n/a
03030	DNA replication	50	23	68%
03420	Nucleotide excision repair	48	22	100%
04010	MAPK signaling pathway	177	33	51%
04020	Calcium signaling pathway	130	21	100%
04120	Ubiquitin mediated proteolysis	116	45	100%
04310	Wnt signaling pathway	100	18	28%
04810	Regulation of actin cytoskeleton	133	35	41%
04110	Cell cycle	91	29	n/a
04510	Focal adhesion	125	39	59%
04720	Long term potentiation	42	14	83%

a Data on number of KEGG Enzymes by Gene in each pathway from http://www.genome.jp/dbget-bin/get_linkdb?pathwaymap00010

b This reflects the percent coverage in the core pathway reactions, but not all of the side-entry or alternative metabolite routes into the pathway. Data presented for pathways where counting the number of core reactions was possible. In the case of signaling pathways, or multicomponent enzyme systems (e.g., E3 ubiquitin) the number of categories, but not individual components, for which our library covers are given. When prokaryotic and eukaryotic pathways are both presented in the KEGG pathway map, we have only included the eukaryotic set here. Due to complexity of pathways 03022 and 04110, the percentage coverage of these KEGG maps has not been calculated.

**Figure 4:**
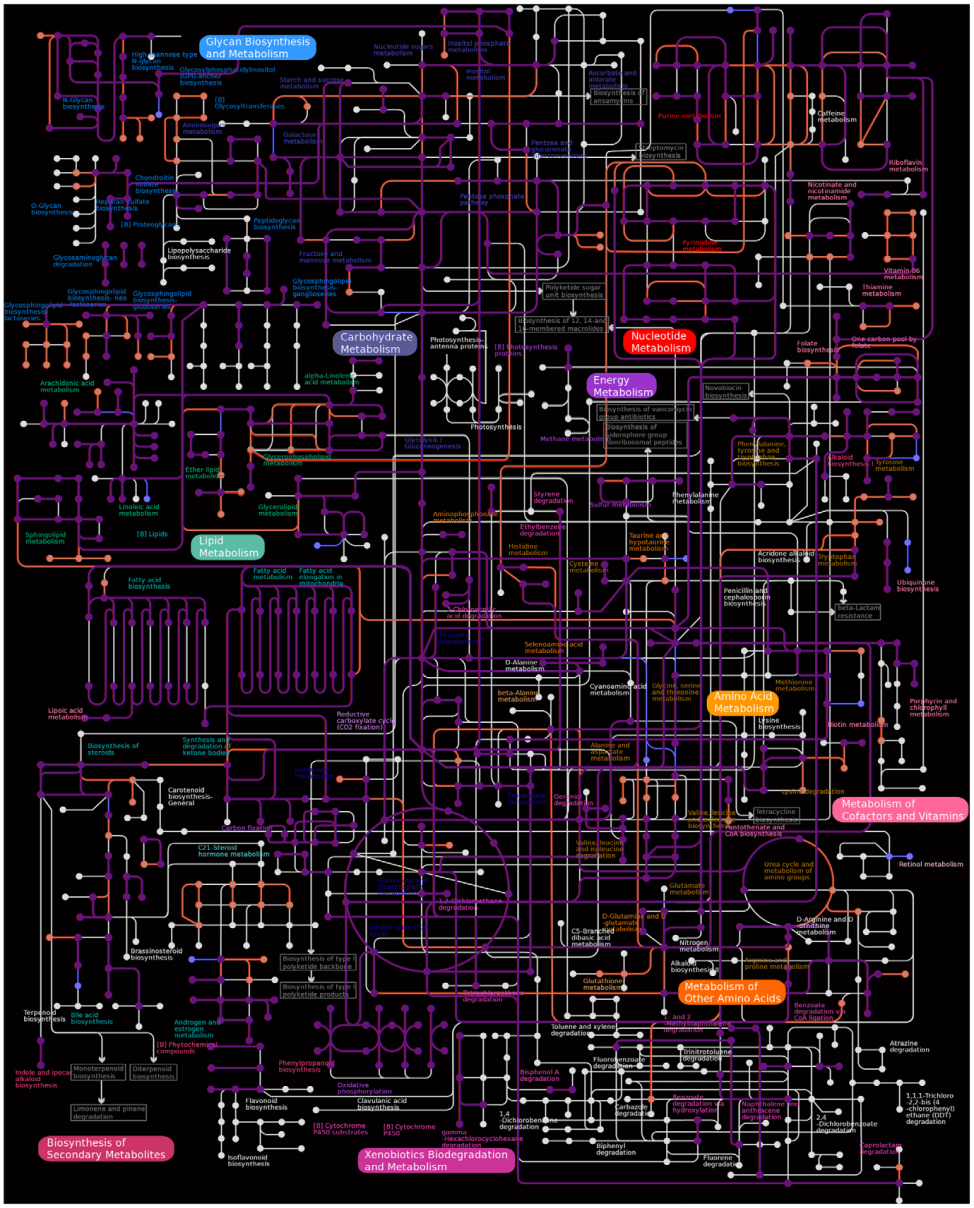
Overlay of metabolic pathway map generated by the KEGG KAAS for both the *Petrolisthes cinctipes* and *Daphnia pulex* analyses. Enzymes are represented by lines, and compounds are represented by dots. Coloration indicates that genes were found in both both *P. cinctipes* and *D. pulex* (purple), only *P. cinctipes* (blue), only *D. pulex* (red), or in neither of the data sets (white).

Graphic comparison of coverage of central metabolic pathways in the porcelain crab transcriptome compared to coverage resulting from the *Daphnia pulex* genome project is a useful mechanism for assessing the completeness of the *Petrolisthes cinctipes* transcriptome. While the porcelain crab ESTs we have generated have excellent coverage of carbohydrate and energy metabolism (Figure 4, purple lines), they lack in coverage of nucleotide metabolism, and to a lesser extend lipid and amino acid metabolism (Figure 4, red lines). For example, several glycosphingolipid biosynthesis pathways and glycerophospholipid metabolism are much better represented in the *D. pulex* genome than the *P. cinctipes* ESTs (Figure 4). Glycosphingolipids contain the amino alcohol sphingosine, which is a component of sphingomyelin, a lipid component of myelin sheath Schwann cells [36]. Potentially the presence of these pathways in *Daphnia* and the absence in *Petrolisthes* indicates that *Daphnia* have greater potential for possessing myelinated axons, which are known in fast swimming copepods [37], [38]. Several metabolic enzymes were present in the *P. cinctipes* EST set that are not present in the KEGG KAAS metabolic maps from the *D. pulex* genome data (Figure 4, blue lines). Quite likely, the *P. cinctipes* pathway map is an incomplete representation of what the genome holds, and the pathway maps should also be updated as the *D. pulex* genome annotation progresses.

**Figure 5:**
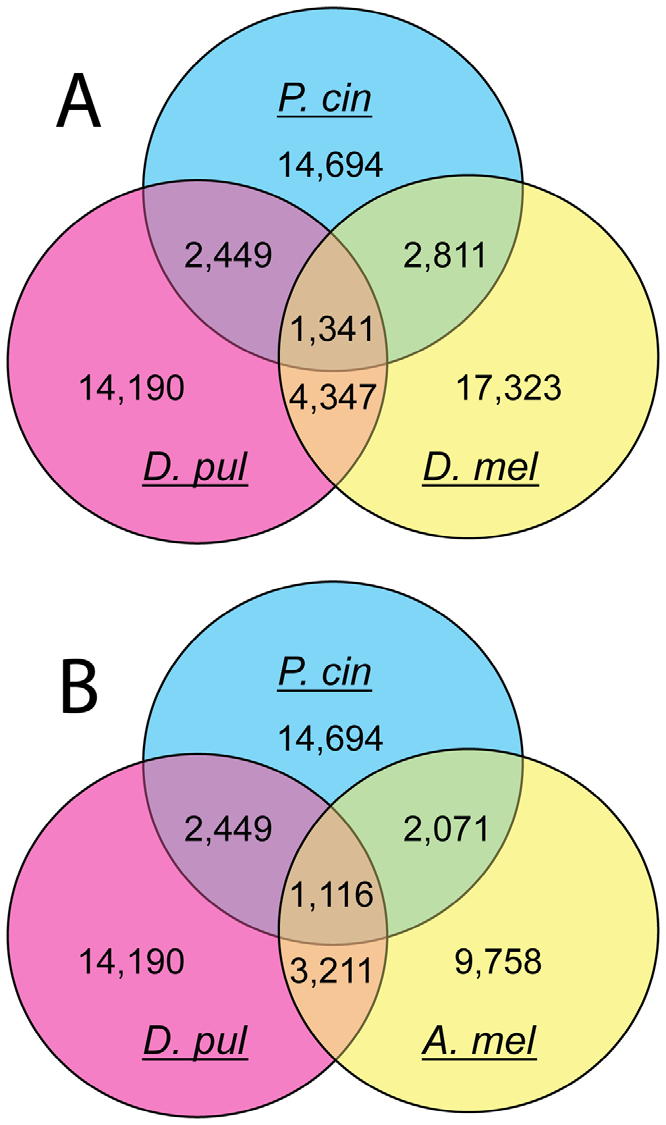
Venn diagram representations of 3-way reciprocal tBLASTx results between porcelain crabs, Daphnia and insects. Comparisons were between unigene sets of *Petrolisthes cinctipes* (P. cin, blue), *Daphnia pulex* (D. pul, pink) and either (A) *Drosophila melanogaster* (D. mel, yellow) or (B) *Apis mellifera* (A. mel, yellow).

Reciprocal 3-way BLASTx analyses between unigene contig sets of *P. cinctipes*, *D. pulex* and either *D. melanogaster* or *A. mellifera* suggest that there is greater homology between *D. pulex* and hexapoda than between *D. pulex* and malacostracan crustacea (Figure 5). In these analyses *P. cinctipes* and *D. pulex* shared fewer common strong homologs than *D. pulex* did with either of the insect taxa. Thus our data support the hypothesis that Branchiopod crustaceans are more closely related to Hexapoda than Malacostracan crustaceans, and agree with phylogenetic reconstructions of all ecdysozoa based on nuclear genes, 18S and 28S ribosomal gene sequences [8].

### PCAD: The Porcelain Crab Array Database

The Porcelain Crab Array Database, PCAD (http://array.sfsu.edu), is a feature-enriched version of the Longhorn Array Database (LAD), which is an open source version of the Stanford Microarray Database (SMD). PCAD uses the operating system Red Hat Enterprise Linux WS release 4, PostgreSQL 7.4.17 as database management system, Apache 1.3.27 as the web server, and Perl 5.8.5 as the development language. In PCAD we integrated an EST sequence and homology database. For each cloned cDNA, PCAD archives each of the ESTs and assembled cluster_consensus and contig sequences in FASTA format on PCAD. Links to GenBank are provided for the EST sequences. Also, for each EST the results of all homology searches are stored, with the top 1–5 hits from BLAST analyses that had e-value scores of ≤1e^−5^, or for the case of InterProScan results, any hits recorded. For each CloneID, a separate set of GO and EC terms generated in InterProScan and KEGG analyses are also provided. For each CloneID, a summary field (“PCAD summary”) has been entered automatically when BLASTx vs. SwissProt returned a strong match. The PCAD summary field is manually curated and each value also has a score of strong, weak or no match depending on homology search results. Our sequence database is searchable by CloneID, by consensus sequence ID, by text search terms (e.g., “lactate dehydrogenase”) or by a keyfile of CloneID names.

We have integrated the sequence database with the microarray data so that each microarray feature is linked to the sequence database for the corresponding CloneID. Microarray data analysis tools of PCAD have been enhanced from those provided in the SMD/LAD architecture to allow display of features representing a selected subset of CloneIDs on a selected subset of microarray slides. In this feature the user can effectively access the raw data in terms of actual feature morphology and intensity for a particular microarray feature across a set of arrays used for a particular analysis. We envision this tool will be particularly useful for assessment of raw data quality following the identification of microarray features that were differentially expressed in a statistical comparison of two or more sample groups.

### Conclusions

With the sequencing of almost 100,000 high-quality ESTs, and their assembly, annotation and incorporation into a novel database named PCAD, we greatly extended the availability of genomic resources for crustaceans, and provided the largest set of sequence data for any species of crab. Comparative genomic analysis using these ESTs to better resolve relationships between crustaceans and hexapod insects leads to the general conclusion that branchiopod crustaceans may be more closely related to insects than other malacostracan crustaceans. The porcelain crab functional genomic resources have been utilized for gene expression microarray development and their application to a variety of questions related to environmental physiology, thermal physiology, cellular physiology, and responses to climate change. We expect this resource to be beneficial for all crustacean biologists, as well as genomics researchers working on insects that require a non-insect arthropod outgroup for their studies. As high-throughput sequencing technology continues to reduce the costs of generation of DNA sequences, we expect the availability of the large transcriptomic dataset presented here to be useful for future genome assembly and gene prediction efforts.

## Supporting Information

Table S1 Full Gene Ontology Results. This spreadsheet lists the full set of gene ontology search results for each of the three ontologies. A summary of this table is represented in Figure 1.
(0.07 MB TXT)
